# Width and neurophysiologic properties of tissue bridges predict recovery after cervical injury

**DOI:** 10.1212/WNL.0000000000007642

**Published:** 2019-06-11

**Authors:** Kevin Vallotton, Eveline Huber, Reto Sutter, Armin Curt, Markus Hupp, Patrick Freund

**Affiliations:** From the Spinal Cord Injury Center (K.V., E.H., A.C., M.H., P.F.) and Department of Radiology (R.S.), Balgrist University Hospital; University of Zurich (K.V., E.H., A.C., M.H., P.F., R.S.), Switzerland; Wellcome Trust Centre for Neuroimaging (P.F.) and Department of Brain Repair and Rehabilitation (P.F.), UCL Institute of Neurology, University College London, UK; and Department of Neurophysics (P.F.), Max Planck Institute for Human Cognitive and Brain Sciences, Leipzig, Germany.

## Abstract

**Objective:**

To assess whether preserved dorsal and ventral midsagittal tissue bridges after traumatic cervical spinal cord injury (SCI) encode tract-specific electrophysiologic properties and are predictive of appropriate recovery.

**Methods:**

In this longitudinal study, we retrospectively assessed MRI scans at 1 month after SCI that provided data on width and location (dorsal vs ventral) of midsagittal tissue bridges in 28 tetraplegic patients. Regression analysis assessed associations between midsagittal tissue bridges and motor- and sensory-specific electrophysiologic recordings and appropriate outcome measures at 12 months after SCI.

**Results:**

Greater width of dorsal midsagittal tissue bridges at 1 month after SCI identified patients who were classified as being sensory incomplete at 12 months after SCI (*p* = 0.025), had shorter sensory evoked potential (SEP) latencies (*r* = −0.57, *p* = 0.016), and had greater SEP amplitudes (*r* = 0.61, *p* = 0.001). Greater width of dorsal tissue bridges predicted better light-touch score at 12 months (*r* = 0.40, *p* = 0.045) independently of baseline clinical score and ventral tissue bridges. Greater width of ventral midsagittal tissue bridges at 1 month identified patients who were classified as being motor incomplete at 12 months (*p* = 0.002), revealed shorter motor evoked potential (MEP) latencies (r = −0.54, *p* = 0.044), and had greater ratios of MEP amplitude to compound muscle action potential amplitude (*r* = 0.56, *p* = 0.005). Greater width of ventral tissue bridges predicted better lower extremity motor scores at 12 months (*r* = 0.41, *p* = 0.035) independently of baseline clinical score and dorsal tissue bridges.

**Conclusion:**

Midsagittal tissue bridges, detectable early after SCI, underwrite tract-specific electrophysiologic communication and are predictors of appropriate sensorimotor recovery. Neuroimaging biomarkers of midsagittal tissue bridges may be integrated into the diagnostic workup, prediction of recovery, and patients' stratification in clinical trials.

MRI is often applied after traumatic spinal cord injury (SCI) to evaluate the level and extent of intramedullary damage.^1^ Serial MRI studies have demonstrated evolving patterns of intramedullary signal changes, ranging from acute changes (e.g., edema/hemorrhage) to subacute changes (e.g., cyst formation) to chronic changes (e.g., cyst collapse and syringomyelia).^2^ Quantification of lesion size shortly after trauma has revealed relationships between lesion severity and clinical impairment at admission and discharge.^1,3–5^ Only after weeks, once edema and hemorrhage have receded, can preserved neuronal tissue be identified adjacent to developing cysts.^2^ This neuronal tissue is the only remaining bridge connecting supralesional and infralesional neuronal networks. Crucially, these tissue bridges underwrite clinically relevant electrophysiologic communication.^2^ Thus, preserved neuronal tissues that can be quantified on midsagittal T2-weighted scans have been called midsagittal tissue bridges.^2^

Midsagittal tissue bridges are typically located both dorsally and ventrally relative to posttraumatic cysts, and their combined widths are predictive of clinical recovery.^2,6^ However, the potential tract specificity of dorsal vs ventral midsagittal tissue bridges is overlooked when their widths are combined. On the basis of the known anatomic topology of the spinal cord, we hypothesized that dorsally located midsagittal tissue bridges should conduct ascending sensory signals, while ventrally located midsagittal tissue bridges should conduct descending motor signals. To validate the tract specificity of dorsal and ventral midsagittal tissue bridges, we investigated associations between their widths and sensory (SEP)^7^ and motor (MEP)^8^ evoked potentials and appropriate recovery. This work evaluates midsagittal tissue bridges as clinically relevant neuroimaging biomarkers that can potentially improve predictions of tract-specific outcomes and the stratification of patients in trials.

## Methods

### Study participants

We retrospectively collected imaging, electrophysiologic, and clinical data for 28 patients with traumatic cervical SCI who were admitted between January 2005 and September 2014 at the University Hospital Balgrist, Zurich, Switzerland. This cohort included a subgroup of patients previously reported in a study focused on the combined widths of tissue bridges.^2^ Patients with cervical SCI and disease duration of no longer than 2 months before the first assessment who took part in a 12-month follow-up assessment were eligible to participate. Patients with brain lesions or preexisting neurologic or medical disorders leading to functional impairment or mental illness were excluded, as well as patients with contraindications to MRI.

### Standard protocol approvals, registrations, and patient consents

The study protocol was approved by the local Ethics Committee (EK-2010-0271), and all patients gave their informed written consent before study enrollment.

### Clinical and functional assessments

Clinical examinations were performed at 28 ± 7 (mean ± SD) and 371 ± 5 days after SCI. Motor and sensory functions were assessed by means of the International Standards for Neurological Classification of SCI,^9,10^ and functional independence was assessed with the spinal cord independence measure (SCIM).^11^ The SCIM score was missing in 1 patient at 1 month after SCI (patient 19) and in another patient at 12 months (patient 26); the light-touch and pinprick scores were also missing in 1 patient at 1 month (patient 11).

### Electrophysiologic recordings and analysis

The electrophysiologic examinations were conducted at 87.19 ± 12 days after SCI according to the standard protocol of the European Multicenter Study About Spinal Cord Injury (EMSCI).^12,13^ To obtain tibial SEPs, posterior tibial nerves were stimulated bilaterally at the ankle. The stimulation was performed until a motor response was induced to ensure that all fibers were stimulated. Cortical responses were recorded with an active electrode at Cz′ (2 cm posterior to Cz) and a reference at Fz according to the 10-2020 EEG system. The impedance was maintained at <5 kΩ. Two sets of 150 responses were averaged and superimposed. The SEP P40 latency was measured as the time from the stimulation to the first positive peak of the primary complex, and the amplitude was measured as the difference between the P40 and N50 (first negative) peaks. SEPs were recordable in 19 patients.

Abductor hallucis MEPs were acquired by single-pulse transcranial magnetic stimulation, placing the coil at 4 cm rostral of Cz, thus provoking an abductor hallucis muscle response. A sample frequency of 2,000 Hz, biphasic stimulus duration of 200 microseconds, and a band-pass filter of 30 Hz to 1 kHz were used. The time from the stimulation to the muscle response onset determined the MEP latency, and the amplitude was measured from baseline to the highest negative peak of the potential. Fifteen patients had recordable MEPs.

To assess peripheral nervous system damage that could interfere with the MEP and SEP results, peripheral motor nerve conduction studies of the tibial nerves were conducted in all patients. The compound muscle action potential (CMAP) amplitude and nerve conduction velocity were quantified after distal and proximal stimulations of the tibial nerves.

The electrophysiologic outcome measures included SEP latency and amplitude, MEP latency, and MEP amplitude/CMAP ratio. The MEP amplitude was normalized by the CMAP ratio, which allowed assessment of only the central component of the MEP independently of the peripheral nervous system.^14^ For all measures, the average of the bilateral response was used for analysis because no lateralization could be distinguished in midsagittal MRI slices. In addition, SEP and MEP latencies were normalized for height.^15^ SEP measurements were missing in 1 patient (patient 19); 7 did not show any answer potential; and 20 had a recordable potential, of whom 19 had reliably measurable P40 latencies. MEP measurements were missing in 2 patients (patients 2 and 19); 17 had a recordable potential; 16 had reliably measurable latencies; and 9 had no recordable answer. One patient (patient 1) was excluded from both SEP and MEP analyses because of pathologic neurography. Patients without any recordable evoked potential were not included in the latency analysis.

### Imaging protocol and image analysis

Patients underwent MRI at 35.3 ± 16 days after SCI. Nine patients were scanned with a 3T Magnetom Verio MRI scanner, 6 with a 3T Magnetom Skyra^fit^ scanner, 4 with a 1.5T Magnetom Symphony, 5 with a Magnetom Espree, 3 with a Magnetom Avanto (all Siemens Healthcare, Munich, Germany), and 1 with a Signa HDx (GE Medical Systems, Dallas, TX). A 16-channel receive head and neck coil was used with all scanners.

The MRI protocol included axial T2-weighted and sagittal T1- and T2-weighted sequences (1.5T: repetition time 4,057 milliseconds, echo time 113 milliseconds, flip angle 146°; 3T: repetition time 3,327 milliseconds, echo time 82 milliseconds, flip angle 151°); the measurements of tissue bridges were performed only on the T2-weighted midsagittal slice.^2^

**Figure 1:**
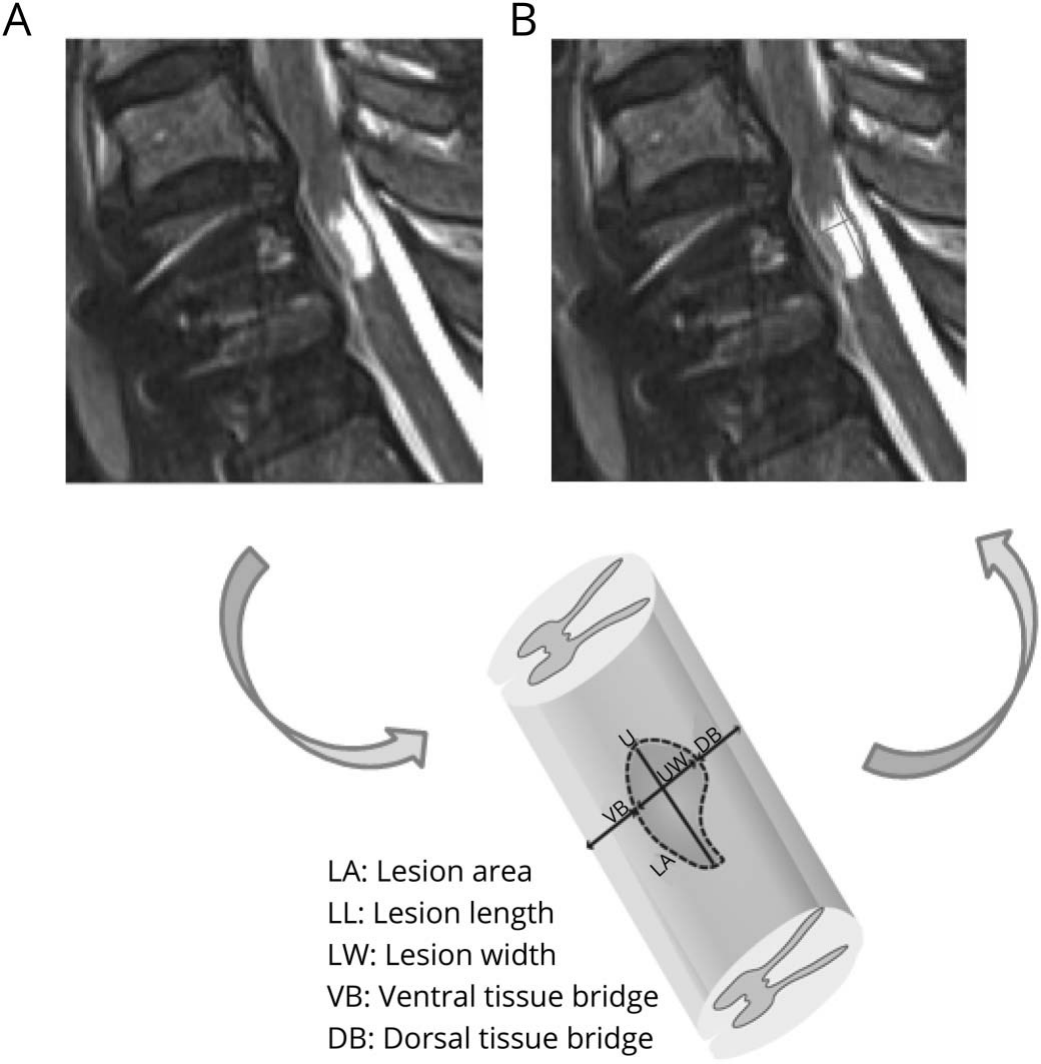
Segmentation of the midsagittal tissue bridges on MRI (A) T2-weighted midsagittal slice showing a hyperintense intramedullary signal at the lesion epicenter at 1 month after spinal cord (SC) injury. (B) Schematic showing how the lesion, SC borders, and anterior-posterior widths of the ventral (shortest distance from anterior SC border to lesion) and dorsal (shortest distance from posterior SC border to lesion) tissue bridges were segmented manually.

Before lesions and midsagittal tissue bridges were identified and quantified, an experienced radiologist (R.S.) screened for the presence of edema or hemorrhage. Lesions were identified as hyperintense signals on T2-weighted images.^16^ Jim 6.0 software (Xinapse Systems, Aldwincle, UK) enabled semiautomatic lesion delineation and measurement of the anterior-posterior width of dorsal and ventral midsagittal tissue bridges^2^ (figure 1). The lesion analysis was blinded for patients' clinical and electrophysiologic data. Intraobserver and interobserver reliabilities are high, as previously reported.^2^

### Statistical analysis

We used Stata 14 (StataCorp LP, College Station, TX) for statistical analysis. We tested clinical recovery over 12 months after SCI via a paired *t* test. We investigated whether the width of midsagittal tissue bridges differed in patients with sensory or motor completeness at 12 months after SCI compared to incomplete patients using a *t* test. Regression analysis assessed associations between MRI measurements and electrophysiologic data. In these models, SEP or MEP was entered as the response variable, and the width of the ventral or dorsal midsagittal tissue bridges was entered as a predictor. These regression models were corrected for age, sex, and the tissue bridge of no interest (ventral midsagittal tissue bridges for SEPs and dorsal midsagittal tissue bridges for MEPs). These potential confounders were entered singly into models to keep the number of covariates per model to an absolute minimum and were retained only if there was a material impact on the regression coefficient of interest or the covariate was significant. The partial correlation coefficients from this regression analysis are reported in the results. We used regression models to assess whether the widths of dorsal and ventral midsagittal tissue bridges were associated with specific sensory and motor clinical recovery at 12 months after SCI, correcting for age, sex, and 1-month clinical scores. We also used regression analysis to investigate whether electrophysiology was predictive of clinical outcome, correcting for age and 1-month clinical score.

### Data availability

Anonymized data not published within this article will be made available by request from any qualified investigator.

## Results

### Clinical, electrophysiologic, and radiologic characteristics

Twenty-eight patients with a traumatic cervical SCI <2 months before study enrollment who had a 12-month follow-up assessment were recruited. Five patients were classified as complete (American Spinal Injury Association Impairment Scale [AIS] score A) and 23 as incomplete (AIS grade B–D) at 1 month after SCI (table 1). The neurologic level of SCI varied between C1 and C7 but was predominantly at C4 (32%) and C3 (29%). At 12 months after SCI, 5 patients had recovered 1 AIS grade and 1 patient had recovered 2 AIS grades. Over the 12 months after SCI, patients recovered by 7 ± 8 points (from 23 to 30 points) on the lower extremity motor score (LEMS) (*p* < 0.0001, n = 28), by 8 ± 15 points (from 66 to 74 points) on the light-touch score (*p* = 0.0061, n = 27), by 8 ± 21 points (from 55 to 63 points) on the pinprick score (*p* = 0.0254, n = 17), and by 36 ± 30 (from 28 to 64 points) on the SCIM score (*p* < 0.0001, n = 26) (table 2).

**Table 1:**
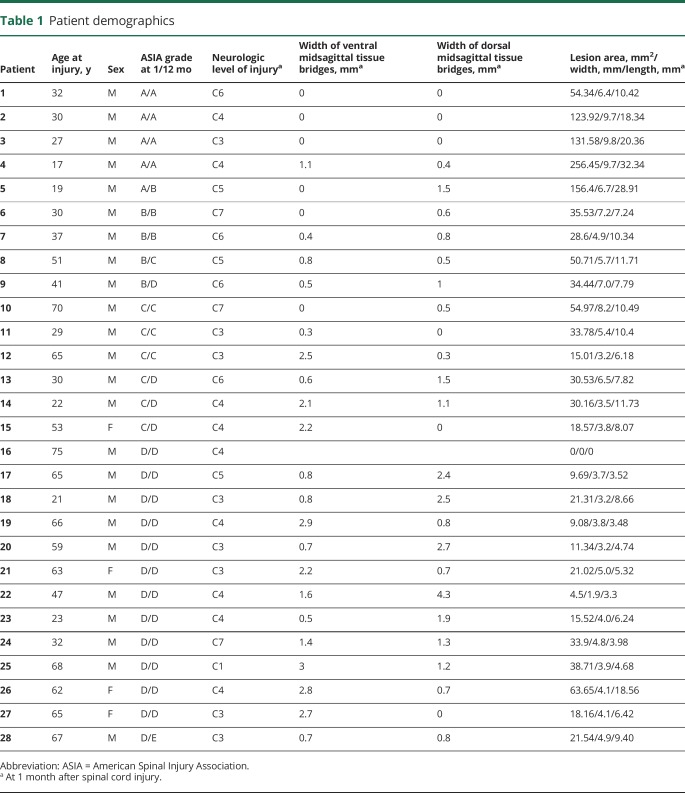
Patient demographics

**Table 2:**
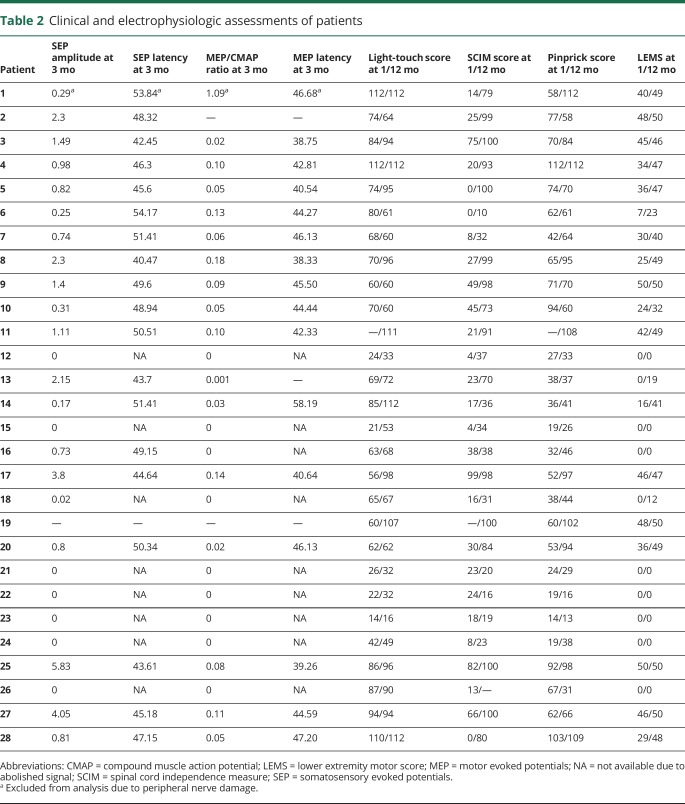
Clinical and electrophysiologic assessments of patients

The mean CMAP of the tibial nerves was 9.39 ± 4.25 mV, and the mean nerve conduction velocity was 45.99 ± 4.68 m/s. One patient (patient 1) had a CMAP <1 mV, which indicates relevant peripheral neuronal damage. Thus, this patient was excluded from further electrophysiologic analysis.

The mean SEP amplitude was 1.58 ± 1.5 µV (n = 19), and the mean P40 latency was 47.39 ± 3.6 milliseconds (n = 18). The mean MEP amplitude was 0.74 ± 0.6 mV (n = 16), and the mean MEP latency was 43.87 ± 4.9 milliseconds (n = 15) (table 2).

Signs of edema were found in 16 patients, and signs of hemorrhage were seen in 5 patients. Twenty-five of 28 patients had midsagittal tissue bridges, of whom 19 had both dorsal and ventral midsagittal tissue bridges. In addition, 3 patients had dorsal midsagittal tissue bridges only, while another 3 patients had ventral midsagittal tissue bridges only. The average widths of ventral and dorsal midsagittal tissue bridges were 1.1 ± 1.0 and 1.0 ± 1.0 mm (table 1), respectively. In 3 of 5 patients with an AIS grade of A, parasagittal but no midsagittal tissue bridges were detectable. The remaining 2 patients with an AIS grade of A had widths of ventral midsagittal tissue bridges below the study population average (0.5 mm) but widths of dorsal midsagittal tissue bridges (0.95 mm) within the range of incomplete patients. One of those 2 patients converted from AIS grade A to B at 12 months after SCI.

### Relationships between dorsal midsagittal tissue bridges, SEPs, and sensory recovery

**Figure 2:**
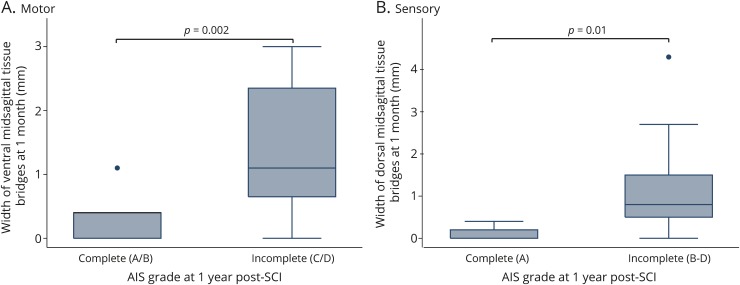
Relationships between midsagittal tissue bridges and ASIA classification (A) Boxplot showing the widths of dorsal midsagittal tissue bridges at 1 month after spinal cord injury (SCI) for patients classified as being sensory complete and incomplete at 12 months after SCI. Note that in the left-hand boxplot, no whisker was shown because all values were between the 25th and 75th percentiles. Sensory complete = American Spinal Injury Association (ASIA) Impairment Scale (AIS) grade A. Sensory incomplete = AIS grades B through D. (B) Boxplot showing the widths of ventral midsagittal tissue bridges at 1 month for patients classified as being motor complete and incomplete at 12 months after SCI. Motor complete = AIS grade A and B. Motor incomplete = AIS grade C and D. Note that in all the boxplots shown in A and B, the lower boundaries of the boxes indicate the 25th percentile, a dark line within each box marks the median, and the upper boundaries of the boxes indicate the 75th percentile. Whiskers above and below the box indicate the 10th and 90th percentiles. Points above the upper whisker indicate outliers above the 90th percentile.

Patients classified as being sensory incomplete (AIS grade B–D) at 12 months after SCI already had wider dorsal midsagittal tissue bridges at 1 month compared to sensory complete patients (AIS grade A) (n = 27, *p* = 0.025; AIS A 0.1 ± 0.2 mm, AIS B–D 1.2 ± 1 mm) (figure 2A).

**Figure 3:**
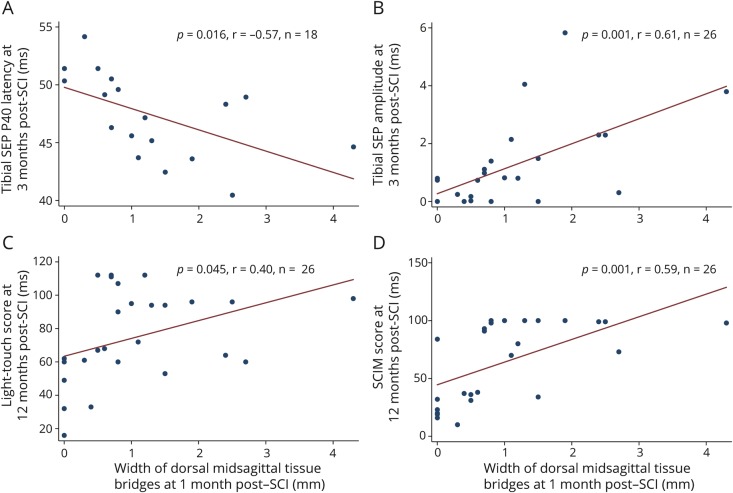
Relationships between dorsal midsagittal tissue bridges and electrophysiologic parameters and clinical outcomes Correlation between dorsal midsagittal tissue bridge width at 1 month and (A) tibial sensory evoked potential (SEP) P40 latency and (B) tibial SEP amplitude at 3 months after spinal cord injury (SCI). Correlation between dorsal midsagittal tissue bridge widths at 1 month (C) and light-touch scores and (D) spinal cord independence measure score at 12 months after SCI. Red line represents the fitted values.

Shorter SEP latencies (*r* = −0.57, *p* = 0.016, n = 18) and higher SEP amplitudes (*r* = 0.61, *p* = 0.001, n = 26) were observed in patients with wider dorsal midsagittal tissue bridges independently of ventral midsagittal tissue bridge width (figure 3, A and B).

Wider dorsal midsagittal tissue bridges at 1 month were associated with better light-touch scores at 12 months (*r* = 0.40, *p* = 0.045, n = 26, figure 3C) independently of 1-month light-touch score, ventral midsagittal tissue bridge widths, age, and sex. Wider dorsal midsagittal tissue bridges at 1 month were also associated with higher SCIM scores at 12 months independently of 1-month SCIM score, age, and sex (*r* = 0.59, *p* = 0.001, n = 26, figure 3D). Dorsal tissue bridge width was not associated with pinprick scores at 12 months (*r* = 0.16, *p* = 0.430, n = 26).

Shorter SEP latency was associated with better light-touch score at 12 months after SCI independently of 1-month light-touch score, age, and sex (*r* = −0.56, *p* = 0.024, n = 17). Better light-touch scores at 12 months after SCI were also found in patients having higher SEP amplitudes (*r* = 0.45, *p* = 0.022, n = 26), but not if the 1-month light-touch score was included in the model (*r* = 0.30, *p* = 0.15, n = 25).

### Relationships between ventral midsagittal tissue bridges, MEPs, and motor recovery

Patients classified as being motor incomplete (AIS grade C–D) at 12 months after SCI had wider ventral midsagittal tissue bridges at 1 month compared to motor complete patients (AIS grade A–B) (n = 27, *p* = 0.002, AIS grade A–B 0.21 ± 0.4 mm; AIS grade C–D 1.46 ± 1 mm, figure 2B).

Patients with wider ventral midsagittal tissue bridges had shorter MEP latencies (*r* = −0.54, *p* = 0.044, n = 15) and higher MEP/CMAP amplitude ratios (*r* = 0.56, *p* = 0.005, n = 25).

Wider ventral midsagittal tissue bridges were associated with better LEMS at 12 months independently of LEMS at 1 month, dorsal midsagittal tissue bridge width, age, and sex (*r* = 0.41, *p* = 0.035, n = 27). Ventral midsagittal tissue bridge width at 1 month was associated with higher SCIM score at 12 months independently of 1-month SCIM score, age, and sex (*r* = 0.44, *p* = 0.028, n = 25). No associations were found between ventral midsagittal tissue bridge width at 1 month and pinprick score at 12 months (*r* = 0.31, *p* = 0.134, n = 26).

Higher MEP/CMAP ratio was associated with better LEMS at 12 months after SCI (*r* = 0.68, *p* < 0.0001, n = 25). However, its significance did not survive when adjusted for 1-month LEMS, age, and sex (*r* = 0.37, *p* = 0.085, n = 25).

## Discussion

This study shows that preserved dorsal and ventral midsagittal tissue bridges underwrite tract-specific electrophysiologic communication after cervical SCI. Crucially, the distinction between dorsal and ventral midsagittal tissue bridges can serve as sensory- and motor-specific predictor of recovery. Thus, these midsagittal tissue bridges can supplement predictions based on clinical and electrophysiologic measures alone.

MRI assessments of midsagittal tissue bridges were carried out as early as 1 month after SCI. By this time, the lesion borders were already clearly identifiable and the ventral and dorsal midsagittal tissue bridges were reliably quantifiable, despite some remaining signs of edema and hemorrhage.^2^ The summed widths of preserved midsagittal tissue bridges and their ratio to the spinal cord diameter were shown to be predictors of recovery.^2,6^ However, we demonstrate here that dorsal and ventral midsagittal tissue bridges can serve as sensory- and motor-specific predictors of recovery, respectively. As expected, patients with more severe injuries had narrower dorsal and ventral midsagittal tissue bridges. The size and location of preserved midsagittal tissue bridges were independent of age and sex. This suggests that the main determinants of preserved tissue bridges are the injury mechanisms (i.e., compression, rotation, flexion-extension) rather than the patients' demographics.

Within the sensory system, we found a 3-way relationship between the width of dorsal midsagittal tissue bridges (i.e., dorsal columns), SEPs, and sensory recovery. Dorsal column function is crucial for proprioceptive recovery.^17–20^ The large and fast-conducting Aβ fibers of the dorsal columns are responsible for touch sensation^21^ and are the anatomic substrate of SEPs.^7^ Because these fibers are particularly vulnerable, as a result of their high demand in trophic support,^22^ SEP measures can be used to assess the severity of damage in the sensory and proprioceptive system after SCI.^23^ SEP latencies are determined mostly by the extent of myelination and can be altered in SCI due to demyelination and impaired remyelination.^24,25^ The amplitude of the evoked potentials reflects the number of synchronously activated axons and their excitability.^26^ Therefore, these measures are sensitive to the number of preserved fibers in the dorsal columns. In our cohort, shorter SEP latencies and higher amplitudes were found in patients having wider dorsal midsagittal tissue bridges. This suggests that wider dorsal midsagittal tissue bridges contain more myelinated axons that are able to conduct ascending information within the dorsal columns. There is a general consensus that functional recovery depends on the number^27,28^ and location^28,29^ of remaining fibers bridging the lesion site. The width of dorsal midsagittal tissue bridges at 1 month was associated with sensory incompleteness and with the extent of epicritic sensory recovery at 12 months independently of the width of ventral midsagittal tissue bridges and baseline clinical score. Moreover, protopathic sensory recovery (i.e., pinprick) was not associated with the width of dorsal midsagittal tissue bridges. These findings highlight the specificity of preserved dorsal tissue bridges for epicritic sensory recovery.

Within the motor system, we found a 3-way relationship between ventral midsagittal tissue bridges, MEPs, and motor recovery of the lower limbs. The MEP assessments quantify noninvasively the cortical and spinal excitability of monosynaptic (i.e., corticospinal tract [CST]^30^) and arguably the polysynaptic (i.e., extrapyramidal) pathways.^31^ After SCI, increases in MEP amplitudes over time have been shown to predict recovery of lower limb function.^32^ Thus, the CST is crucially involved in these recovery processes. However, in animal models of SCI, the contribution of polysynaptic pathways to recovery processes has been suggested.^33,34^ For instance, plasticity in the cortico-reticulo-spinal circuit, which is in part ventrally located in the spinal cord, promoted recovery of locomotion in a rodent model of SCI.^35^ Other potential compensatory mechanisms of motor recovery include sprouting of the anterior CST^36^ and the formation of detour pathways via long-projecting propriospinal neurons.^37–40^ Our findings support the role of the anterior CST in motor recovery processes after SCI in that we found associations between ventral midsagittal tissue bridges, MEP measurements, and improved motor outcomes in the lower limbs. Moreover, only ventral, but not dorsal, midsagittal tissue bridges were predictive of motor recovery. Thus, pyramidal fibers (i.e., anterior CST) running through ventral midsagittal tissue bridges may be responsive to MEP activity and may contribute, along with extrapyramidal fibers (i.e., the ventral part of the cortico-reticulo-spinal tract) to motor recovery, especially if the function of the lateral CST is obliterated by trauma. Thus, the plasticity of the anterior CST^36,37,41^ and possibly of the extrapyramidal tracts^35^ may be important in recovery of motor function after SCI in humans.

Finally, patients' functional independence depends on both motor and sensory functions.^42,43^ The importance of dorsal column integrity is illustrated by the fact that SEP measurements immediately after injury predict recovery of proprioception, an important function for locomotion^44^ and dexterity.^45^ As expected, we found associations between both ventral and dorsal midsagittal tissue bridges and functional independence (i.e., activities of daily living [SCIM score^11^]), emphasizing the importance of both motor and sensory functions to achieve higher levels of functional independence.

Our study has some limitations. As a retrospective case-series study, it may have suffered from selection bias because only traumatic tetraplegic patients with predefined inclusion criteria from 1 center were recruited. Although this resulted in a more homogeneous dataset, reducing interparticipant variance, it might not be entirely generalizable to the SCI population. However, the demographic distribution of our cohort (e.g., mean age of 45 years, the high male/female ratio, and severity scales ranging from mild to severe) is representative of the general SCI population with a cervical injury.^46^ Tissue bridges could not be assessed on T2-weighted axial slices because of their low spatial resolution. However, the assessment of tissue bridges on the midsagittal slice has high intraobserver and interobserver reliabilities,^2^ making it a clinical feasible assessment tool. In our center, the timing of clinical assessments follows the standards of the international EMSCI study^13^ with longitudinal clinical, electrophysiologic, and MRI assessments obtained at 4 time points spread over the first year after SCI (i.e., at admission and 3, 6, and 12 months).^13^ While we chose to use the clinical and MRI assessments at baseline, the electrophysiologic recordings were derived from the 3-month time point. This was motivated by the fact that at this time point the electrophysiologic recordings have recovered without further significant changes over time.^28,37^ Hence, the associations between tissue bridges and clinical outcomes would not be influenced by a change in electrophysiologic parameters. Note that not all patients had MEP and SEP potentials, thus reducing the number of observations in some regression analyses. However, in all regression analyses, sufficient numbers of patients (≥15) per variable were included. Finally, we did not adjust for multiple comparisons because we are investigating a number of different hypotheses, and in such a context, multiple comparison correction can be inappropriate.^47–50^ Nevertheless, as always, there is a risk of spuriously significant results, and *p* values close to 0.05 should be interpreted cautiously and regarded as hypothesis-generating results to be examined in future studies.

This study shows that the widths of dorsal and ventral midsagittal tissue bridges measured as early as 1 month after SCI are in vivo predictors of tract-specific long-term functional recovery. Crucially, the 3-way association between clinical outcomes, widths of ventral and dorsal midsagittal tissue bridges, and electrophysiologic integrity speaks to the clinical relevance of the neuroimaging biomarkers. Therefore, we suggest integrating these neuroimaging biomarkers into diagnostic workups, prognosis, patient stratification, and clinical trials in both the acute and chronic phases.

## Glossary

AIS: American Spinal Injury Association Impairment Scale
CMAP: compound muscle action potential
CST: corticospinal tract
EMSCI: European Multicenter Study About Spinal Cord Injury
LEMS: lower extremity motor score
MEP: motor evoked potential
SCI: spinal cord injury
SCIM: spinal cord independence measure
SEP: somatosensory evoked potential
